# Efficacy and tolerability of quetiapine versus haloperidol in first-episode schizophrenia: a randomized clinical trial

**DOI:** 10.1186/1755-7682-6-47

**Published:** 2013-12-05

**Authors:** Mostafa Amr, Shaheen E Lakhan, Sarila Sanhan, Dahoud Al-Rhaddad, Moussa Hassan, Mohamed Thiabh, Tarek Shams

**Affiliations:** 1 Mansoura University, Mansoura, Egypt; 2 Global Neuroscience Initiative Foundation, Beverly Hills, CA, USA; 3 Neurological Institute, Cleveland Clinic, Cleveland, OH, USA; 4 National Center for Health, Amman, Jordan; 5 9500 Euclid Ave, S100A, 44195 Cleveland, OH, USA

## Abstract

**Background:**

Schizophrenia is a chronic disease of global importance. The second-generation antipsychotic quetiapine has a favorable side-effect profile, however, its clinical effectiveness has been called into question when compared with first-generation antipsychotics such as haloperidol. This study evaluates the efficacy and tolerability of quetiapine versus haloperidol for first-episode schizophrenia in the outpatient setting.

**Methods:**

156 adult patients with first-episode schizophrenia participated in an outpatient clinical trial and were randomized to quetiapine (200 mg/d; n = 78) or haloperidol (5 mg/d; n = 78). The study medications were titrated to a mean daily dose of 705 mg for quetiapeine and 14 mg for haloperidol. The patients were assessed at baseline, six weeks, and twelve weeks. The primary outcome measures were positive and negative scores of the Positive and Negative Syndrome Scale (PANSS). Secondary measures were Global Assessment of Functioning (GAF) scale for overall psychosocial functioning, and Simpson-Angus Scale (SAS) for extra-pyramidal symptoms.

**Results:**

At twelve weeks, the quetiapine group had a greater decrease in PANSS positive (18.9 vs. 15.3, p = 0.013) and negative scores (15.5 vs. 11.6, p = 0.012), however, haloperidol showed a greater decrease in general psychopathology score (23.8 vs. 27.7, p = 0.012). No significant difference between groups were found for total PANSS (58.3 vs. 54.8, p = 0.24) and GAF (45.7 vs. 46.2, p = 0.79).

ANOVA identified significant group interactions on PANSS positive (F = 18.72, df = 1.6,52.4, p < 0.0001), negative (F = 5.20, df = 1.1,35.7, p < 0.0001), depression/anxiety (F = 106.49, df = 1.14,37.8, p < 0.0001), and total scores (F = 7.51, df = 1.4,45.6, p = 0.001).

SAS (8.62 vs. 0.26, p < 0.0001) and adverse events of akathisia (78% vs. 0%, p = 0.000), parkinsonism (66.6% vs. 0%, p < 0.0001), and fatigue (84.6% vs. 66.6%, p = 0.009) were greater in haloperidol compared to quetiapine, whereas headache was more common in quetiapine treated patients (11.5% vs. 35.9%, p < 0.0001).

**Conclusions:**

Quetiapine has greater efficacy for positive and negative symptoms with less extra-pyramidal symptoms than haloperidol when used for first-episode schizophrenia in the outpatient setting.

## Background

Schizophrenia is a chronic illness with a lifetime prevalence of 0.7% in the US. The World Health Organization recognizes schizophrenia as being among the top ten causes of disease-related disability worldwide [1]. It confers not only debilitating health consequences, but also significant socio-economic implications. In 2002, the overall cost associated with schizophrenia in the US was estimated at $62.7 billion [2]. The largest component of this figure is the estimated $32.4 billion of indirect costs associated largely with unemployment, reduced workplace productivity, premature mortality from suicide, and family care-giving. Although there is no cure, schizophrenia is highly treatable. Optimal treatment not only reduces the burden of these indirect costs but can improve patient outcomes.

Current evidence-based pharmacological therapy for schizophrenia successfully employs the use of antipsychotic medications [3,4]. Drugs referred to as second-generation antipsychotics are antagonists at both dopamine and serotonin receptors in the central nervous system; in contrast, conventional agents act predominantly on dopamine receptors [5]. Some authors have expressed doubt that second-generation antipsychotics offer any advantage beyond improved tolerability [6-8], and therefore argue for the continued use of conventional agents.

Quetiapine is a second-generation antipsychotic that is approved for the treatment of schizophrenia for not only its favorable side-effect profile but also its clinical efficacy [9]. Several short-term studies indicate that quetiapine is more effective than the conventional antipsychotic haloperidol for the treatment of negative schizophrenic symptoms such as withdrawal from social interactions and blunted emotional expression [10]. For positive symptoms such as hallucinations and delusions, similar efficacy was found with quetiapine when compared to haloperidol in pooled analyses of large controlled trials [11,12].

This study was conducted to investigate quetiapine as treatment for first-episode schizophrenia in an outpatient setting. We hypothesized that treatment with quetiapine would be superior to haloperidol in reducing both negative and positive symptoms while minimizing the occurrence of significant drug-associated adverse events.

## Methods

### Setting and participants

Two centers participated in the study: Al-Bashir Hospital and Al-Karama Hospital both in Amman, Jordan. From October 2009 to September 2011, 210 patients with first episode schizophrenia were assessed for eligibility. Eligible patients were aged 18–60 years and met the criteria for schizophrenia according to the Diagnostic and Statistical Manual of Mental Disorders, fourth edition text revision (DSM-IV-TR) [13]. Exclusion criteria included current or past use of antipsychotics for any psychiatric condition; concurrent DSM-IV Axis I diagnosis, DSM-IV Axis II diagnosis of borderline personality disorder or antisocial personality disorder, or substance dependence or abuse; and clinically significant or unstable medical illness.

### Study design

Patients were randomly assigned based on computerized random number generation to treatment with either quetiapine (200 mg/d) or haloperidol (5 m/d). The data management department of the two study centers generated a random allocation sequence, enrolled participants, and assigned participants to interventions. Patients and investigators assessing outcomes were blind to the allocated intervention. The treating psychiatrist was unmasked to the assigned treatment as this reflected routine clinical practice and increased the trial’s external validity.

Psychiatrists at each site adjusted the doses of identical-appearing tablets to maximize clinical benefits and minimize adverse events. The range of permissible doses was 200–800 mg/d quetiapine and 5–15 mg/d of haloperidol.

Co-medication with psychotropic medications was not permitted with the exception of lorazepam and zopiclone. Lorazepam (1–4 mg/d) was administered for insomnia and agitation and zopiclone (3.75–7.5 mg/d) for insomnia. For extra-pyramidal symptoms (EPS), if dose reduction of the study drug did not achieve the desired effect, the anticholinergic biperiden was prescribed (2–8 mg/d). Besides standard clinical management, no additional psychotherapy was performed.

This trial was performed in accordance with the Declaration of Helsinki and subsequent revisions. The protocol was approved by an institutional review board at each of the participating hospitals. Written consent was obtained from each patient or their legal representative before entering the study. Al-Bashir Hospital and Al-Karama Hospital IRBs.

### Procedures

Interviews and chart review were used to assess for age, gender, marital status, education, income, employment status, and duration of illness. Patients were assessed by a treating psychiatrist and independent rater (psychiatrist) at baseline, six weeks, and twelve weeks after starting the study medication with the following instruments: Positive and Negative Syndrome Scale (PANSS) for schizophrenia symptomatology, the Global Assessment of Functioning (GAF) scale for overall psychosocial functioning, and the Simpson-Angus Scale (SAS) for EPS. Furthermore, at each visit, all patients underwent vital signs, physical examination, safety laboratory assessments (fasting glucose, cholesterol, high-density lipoprotein, low-density lipoprotein, triglycerides, and prolactin), and electrocardiogram (ECG).

### Instruments

#### Positive and Negative Syndrome Scale (PANSS)

The PANSS, a semi-structured interview schedule, was adopted to assess psychotic symptoms [14]. The scale constitutes 30 items including seven positive symptom sub-scale items (P1-P7), seven negative symptom sub-scale items (N1-N7), 16 general psychopathology symptom items (G1-G16), and three depression/anxiety symptoms items (G1-G3, G6). Each item is scored from 1–7 by the interviewer based on the presence and severity of symptoms: (1 = absent, 2 = minimal, 3 = mild, 4 = moderate, 5 = moderate severe, 6 = severe, 7 = extreme). The PANSS ratings are based on all information derived from the clinical interview, direct observation of the subjects, and the reports of primary care staff.

#### Global Assessment of Functioning (GAF) scale

The GAF is a method for representing a clinician’s judgment of a patient’s overall level of psychosocial functioning [15]. The GAF requires a clinician to make an overall judgment about a patient’s current psychological, social, and occupational functioning. In the DSM-IV, this rating is made on a scale from 1 to 100 with ratings of 1 to 10 indicating severe impairment and ratings of 91 to 100 indicating superior functioning.

#### Simpson-Angus Scale (SAS)

The SAS is widely used in both clinical and research settings for the assessment of neuroleptic-induced extrapyramidal side-effects. The SAS consists of 10 items, each scored from 0–4. Higher scores indicate more severe symptoms [16].

### Statistical analysis

The data was analyzed per-protocol using Statistical Package for Social Science. Quantitative variables were tested for normal distributions using the Kolmogorov-Smirnov test. The variables were presented as means ± standard deviation (SD), numbers, and percentages. Student t-tests for independent sample and chi-squared tests were used to evaluate possible differences between quantitative and qualitative data respectively. Two-way repeated measures analyses of variance (ANOVA) was used to assess the effects of treatment (haloperidol vs. quetiapine), time, and an interaction between the treatment and time. Statistical significance was set at the 5% level.

## Results

### Demographics

**Figure 1:**
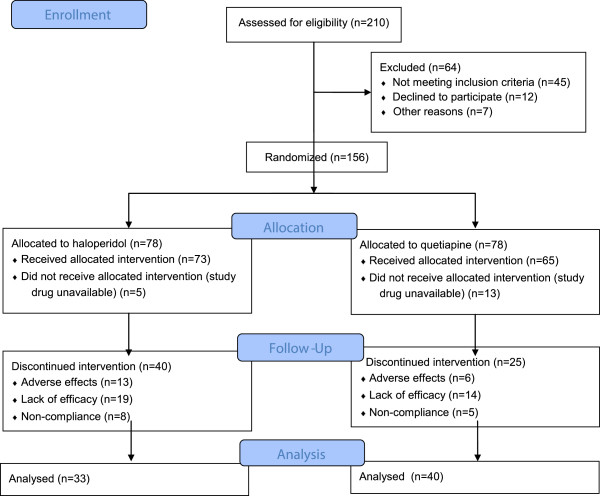
Trial flow diagram demonstrating the disposition of all patients screened for the study.

One hundred and fifty-six total patients with schizophrenia were equally randomized to either quetiapine or haloperidol (Figure 1). The total of 17 patients (5 in the questiapine group and 13 in haloperidol) did not receive the allocated treatment secondary to temporary study-drug unavailability in the hospital pharmacy. Dropout rates were 40/73 (55%) with quetiapine (6 due to adverse effects, 14 to lack of efficacy, and 5 non-compliance) and 25/65 (39%) with haloperidol (13 due to adverse effects, 19 to lack of efficacy, and 8 non-compliance). Thirty-three patients in the quetiapine group and 40 in the haloperidol group completed the twelve-week study.

The patient characteristics of the two analyzed study groups are summarized in Table 1. The two groups were well matched, and there were no statistically significant differences between them regarding the demographic factors, duration of illness, or type of schizophrenia.

**Table 1:** Baseline characteristics of patients analyzed

	**Haloperidol (n = 33)**	**Quetiapine (n = 40)**	**p value**
**Age (years ± SD)**	30.76 ± 3.93	31.29 ± 3.42	0.5396
**Sex (M/F)**	21/12	25/15	0.92
**Duration of illness (months ± SD)**	4.82 ± 1.62	5.03 ± 2.14	0.6438
**Marital status (unmarried/married)**	19/14	23/17	0.994
**Employment status (unemployed/employed)**	22/11	28/12	0.76
**Education (above/below secondary education)**	23/10	31/9	0.449
**Income (satisfactory/unsatisfactory)**	7/26	8/32	0.898
**Type of schizophrenia (paranoid/non-paranoid)**	24/9	32/8	0.464

SD = standard deviation.

### Treatment

The baseline daily doses of quetiapine and haloperidol were 200 mg and 5 mg, respectively. Daily doses of quetiapine and haloperidol were titrated to a mean of 505.8 mg and 12.9 mg by the sixth week and 705.8 mg and 14.2 mg of by the twelfth week, respectively (Tables 2 and 3).

**Table 2:** Common adverse events in all randomized patients

	**Haloperidol (n = 78)**	**Quetiapine (n = 78)**	**p value**
**Akathisia**	53 (78%)	0 (0%)	<0.0001
**Cold**	23 (29.48%)	18 (23%)	0.363
**Headache**	9 (11.5%)	28 (35.9%)	<0.0001
**Fatigue**	66 (84.6%)	52 (66.6%)	0.009
**Parkinsonism**	52 (66.6%)	0 (0%)	<0.0001
**Insomnia**	37 (47.4%)	41 (52.5%)	0.521
**Dizziness**	28 (35.9%)	22 (28.2%)	0.303

**Table 3:** Average daily dose of haloperidol and quetiapine at baseline and after six and twelve weeks

	**Haloperidol (mg)**	**Quetiapine (mg)**
**Baseline**	5.0	200.0
**6 weeks**	12.9 ± 2.49	505.8 ± 101.32
**12 weeks**	14.2 ± 1.79	705.8 ± 101.32

Values given are mean ± standard deviation.

**Table 4:** PANSS, GAF, and SAS at baseline and after six and twelve weeks of treatment

		**Haloperidol (n = 33)**	**Quetiapine (n = 40)**	**t-test**	**p value**
**PANSS positive**	Baseline	23.8 ± 5.12	26.0 ± 4.41	1.90	0.06
	6 weeks	18.2 ± 5.90	21.3 ± 2.51	2.86	0.006
	12 weeks	18.9 ± 7.84	15.3 ± 2.18	2.55	0.013
**PANSS negative**	Baseline	22.2 ± 8.51	21.3 ± 6.38	0.48	0.63
	6 weeks	20.4 ± 8.28	18.9 ± 6.21	0.86	0.39
	12 weeks	15.5 ± 7.39	11.6 ± 4.76	2.59	0.012
**PANSS general psychopathology**	Baseline	39.0 ± 11.01	43.4 ± 8.36	1.939	0.056
	6 weeks	35.1 ± 11.30	37.3 ± 11.01	0.79	0.43
	12 weeks	23.8 ± 6.24	27.7 ± 6.33	2.58	0.012
**PANSS depression/anxiety**	Baseline	10.18 ± 2.11	9.88 ± 1.92	0.6	0.55
	6 weeks	9.88 ± 1.95	9.29 ± 1.64	1.53	0.183
	12 weeks	9.56 ± 1.87	4.74 ± 1.50	11.92	<0.0001
**PANSS total**	Baseline	82.3 ± 21.88	90.8 ± 11.32	1.939	0.056
	6 weeks	73.8 ± 19.50	77.6 ± 8.90	1.02	0.31
	12 weeks	58.3 ± 16.59	54.8 ± 5.93	1.17	0.24
**GAF**	Baseline	32.4 ± 12.03	31.3 ± 7.36	0.47	0.63
	6 weeks	39.5 ± 10.28	40.0 ± 5.69	0.29	0.77
	12 weeks	45.7 ± 9.52	46.2 ± 6.17	0.26	0.79
**SAS**	Baseline	-	-	-	-
	6 weeks	5.94 ± 1.83	0.18 ± 0.38	18.020	<0.0001
	12 weeks	8.62 ± 2.08	0.26 ± 0.45	22.949	<0.0001

Values given are mean ± standard deviation. GAF = Global Assessment of Functioning; PANSS = Positive and Negative symptoms Scale; SAS = Simpson Angus scale.

### Efficacy

**Figure 2:**
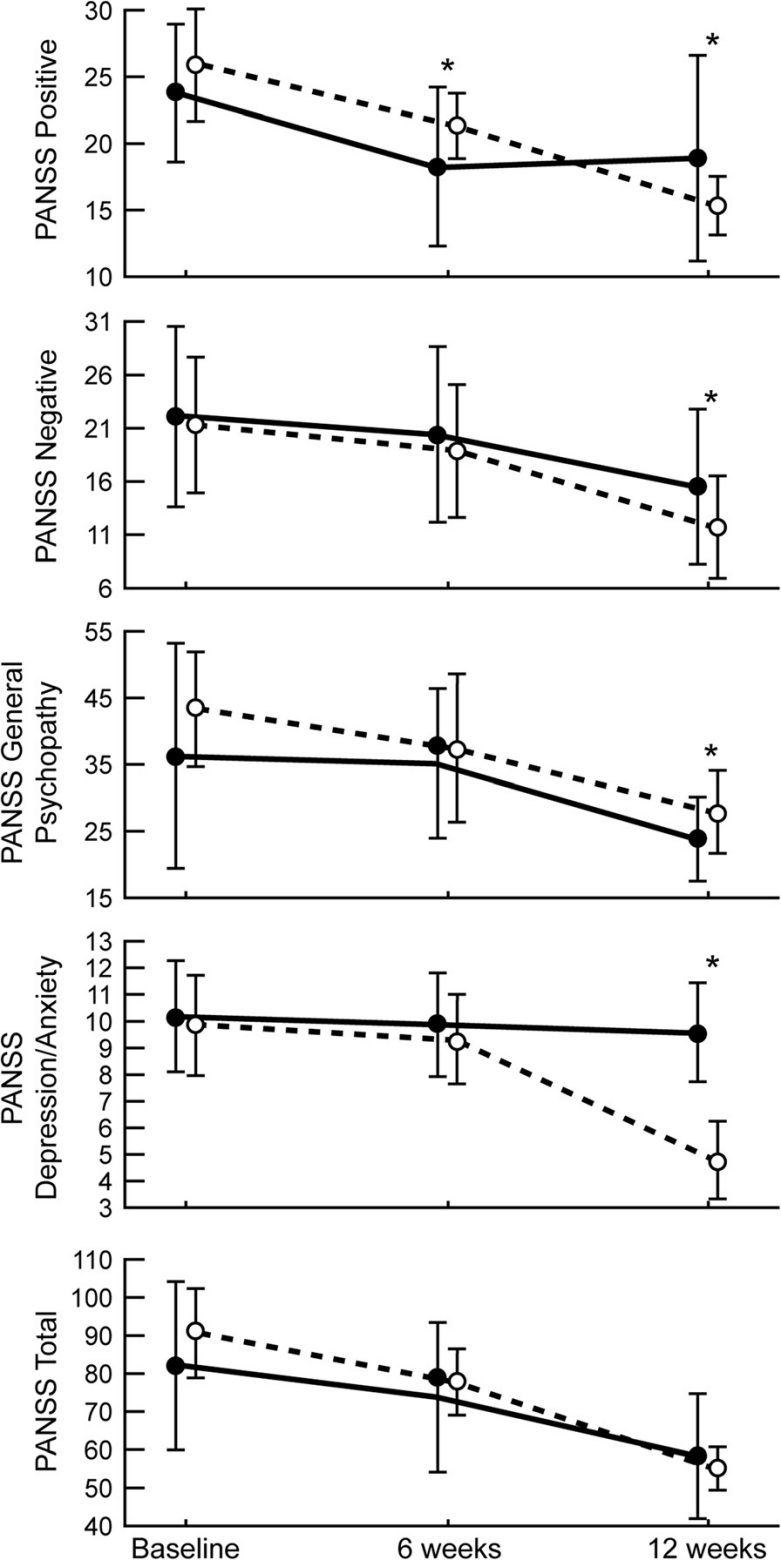
**Mean PANSS at baseline and after six and twelve weeks of haloperidol or quetiapine.** Asterisk indicates statistical significance defined as p < 0.05.

**Figure 3:**
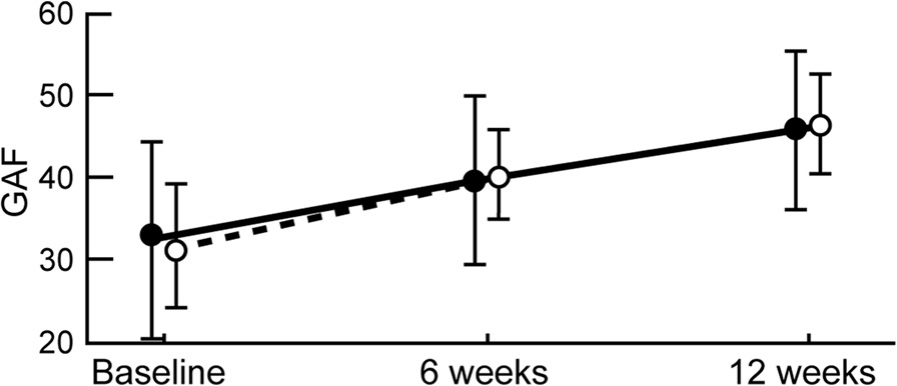
**Mean GAF at baseline and after six and twelve weeks of haloperidol or quetiapine.** Asterisk indicates statistical significance defined as p < 0.05.

**Figure 4:**
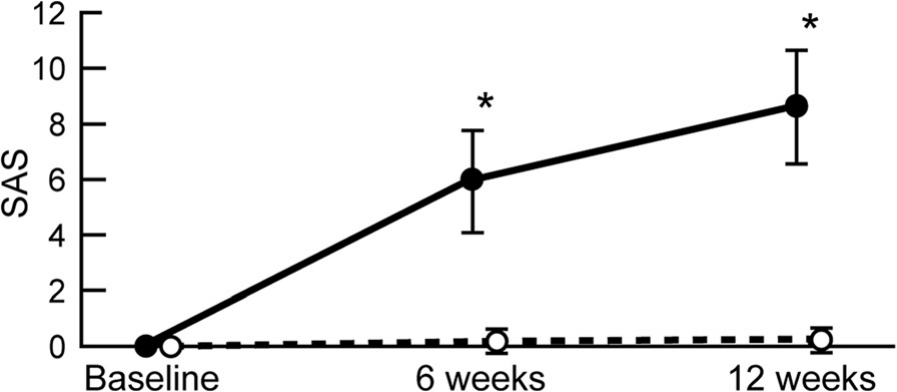
**Mean SAS at baseline and after six and twelve weeks of haloperidol or quetiapine.** Asterisk indicates statistical significance defined as p < 0.05.

The clinical severity of the psychotic symptoms was comparable at baseline and not significantly different across the different scales. As shown in Table 4 and Figures 2, 3, 4, mean scores for PANSS (positive, negative, general psychopathology, depression and anxiety, and total) and GAF improved in both study groups during the trial.

Clinical changes associated with positive symptoms with quetiapine compared to haloperidol were noted at weeks six and twelve, and showed a double phase (Table 4). At six weeks, a significant trend for haloperidol was observed on the PANSS positive score (18.2 ± 5.90 vs. 21.3 ± 2.51, p = 0.006). By the twelfth week, however, quetiapine showed a greater reduction in positive score than haloperidol (18.9 ± 7.84 vs. 15.3 ± 2.18, p = 0.013). By the end of the trial (at the twelfth week), quetiapine showed a significantly greater decrease in negative symptoms than haloperidol as measured by PANSS negative (15.5 ± 7.39 vs. 11.6 ± 4.76, p = 0.012) and depression/anxiety scores (9.56 ± 1.87 vs. 4.74 ± 1.50, p < 0.0001). In contrast, haloperidol showed a significantly greater decrease in PANSS general psychopathology score (23.8 ± 6.24 vs. 27.7 ± 6.33, p = 0.012). However, the effect on GAF (45.7 ± 9.52 vs. 46.26.17, p = 0.798) and total PANSS (58.3 ± 16.59 vs. 54.8 ± 5.93, p = 0.24) were not significantly different.

ANOVA was significantly different on the group effect for general psychopathology and depression/anxiety scores, on the time effect for all PANSS scores and GAF, and on interaction for all the scales with the exception of PANSS general psychopathology score and GAF (Table 5). The most striking effects were observed for the interaction between treatment and time on PANSS positive (F = 18.72, df = 1.6,52.4, p = 0.000), negative (F = 5.20, df = 1.1,35.7, p = 0.000), depression/anxiety (F = 106.49, df = 1.14,37.8, p = 0.000), and total score (F = 7.51, df = 1.4,45.6, p = 0.001). There was no significant interaction effect in PANSS general psychopathology score (F = 0.68, df = 1.1,36.3, p = 0.19) and GAF (F = 1.66, df = 1.23,40.6, p = 0.197).

**Table 5:** Repeated-measures analysis of variance assessments at baseline and after six and twelve weeks of treatment

	**Group effect**			**Time effect**			**Interaction**		
	**F**	**df**	**p**	**F**	**df**	**p**	**F**	**df**	**p**
**PANSS positive**	0.433	1,33	0.51	47.748	1.9,62.2	<0.0001	18.72	1.6,52.4	<0.0001
**PANSS negative**	1.575	1,33	0.21	186.771	1.1,35.7	<0.0001	5.20	1.1,35.7	<0.0001
**PANSS general psychopathology**	16.186	1,33	<0.0001	55.972	1.9,63.7	<0.0001	1.7	1.1,36.3	0.19
**PANSS depression/anxiety**	83.049	1,33	<0.0001	115.78	1.38,45.7	<0.0001	106.49	1.14,37.8	<0.0001
**PANSS total**	1.006	1,33	0.32	186.397	1.4,47.5	<0.0001	7.51	1.4,45.6	0.001
**GAF**	0.000	1,33	0.993	522.74	1.35,44.5	<0.0001	1.66	1.23,40.6	0.197
**SAS**	497.46	1,33	<0.0001	391.60	1.9,62.6	<0.0001	320.43	1.9,63.3	<0.0001

df = degrees of freedom; F = F-test; GAF = Global Assessment of Functioning; p = p-value; PANSS = Positive and Negative symptoms Scale; SAS = Simpson Angus scale.

### Tolerability and adverse events

The prescription rates in the quetiapine and haloperidol groups for lorazepam (2.9 ± 4.5 vs. 3.2 ± 1.7, p = 0.718) and zopiclone (4.6 ± 1.1 vs. 4.3 ± 6.5, p = 0.774) were not statistically different.

Patients treated with haloperidol had significantly higher SAS than quetiapine (5.94 ± 1.83 vs. 0.18 ± 0.38 at six weeks, p < 0.0001; 8.62 ± 2.08 vs. 0.26 ± 0.45 at twelve weeks, p < 0.0001). Accordingly, patients in the haloperidol group required biperiden more often than quetiapine-treated patients (28 vs. 4, p = 0.000). In addition, the dose of biperiden was higher with haloperidol compared with quetiapine (3.45 ± 2.3 vs. 0.21 ± 0.0 mg/d; p < 0.0001).

A single patient in the quetiapine group had a clinically significant laboratory test abnormalities at baseline (mild hypertriglyceridemia). There were no other abnormalities in the vital signs, physical examination, labs, and ECG in either group.

The most frequent adverse events (i.e. with an incidence greater than ten percent) are summarized in Table 2. Akathisia (78% vs. 0%, p < 0.0001), parkinsonism (66.6% vs. 0%, p < 0.0001), and fatigue (84.6% vs. 66.6%, p = 0.009) were higher in the haloperidol group compared to quetiapine. Headache was, however, more common in the quetiapine group than haloperidol (35.9% vs. 11.5%, p < 0.0001).

In the haloperidol group, 13/65 patients (20%) dropped out of the study due to adverse events (7 parkinsonism, 3 akathisia, and 2 fatigue), while 6/73 patients (8.2%) dropped out from quetiapine (4 headache, 1 dizziness, and 1 insomnia).

## Discussion

Significant improvement in the PANSS general psychopathology and depression/anxiety scores were recorded in quetiapine-treated first-episode schizophrenia at twelve weeks. These findings are in contrary to three previous trials that reported no significant difference between groups using PANSS general psychopathology [12,17-19], albeit in study populations not exclusive to first-episode schizophrenia. Purdon and colleagues [19] specifically included patients with schizoaffective disorder and treatment resistance based on history, whereas the others included mixed populations in terms of disorder subtypes and treatment resistance.

Two trials reported no significant difference between haloperidol and quetiapine using Calgary Depression Scale [19,20]. Both studies included mixed populations with respect to disorder subtype and treatment resistance. Daily doses of haloperidol varied from 1–4 mg and 10–20 mg and quetiapine 200–750 mg and 300–600 mg. The duration of follow up was greater than six months in both trials. According to Abou-Setta et al. [21] the risk of bias was high and unclear in these studies. Purdon et al. [19] also reported results for the Beck Depression Inventory (BDI) and found no significant difference. Moreover, a recent meta-analysis examined the efficacy and safety of individual second-generation vs. first-generation antipsychotics in first-episode psychosis and found that quetiapine was similar to haloperidol regarding depression [22].

The tolerability profile of quetiapine was advantageous when compared with haloperidol, as evidenced by the withdrawal rates due to adverse events in each treatment group (8.2% vs. 20%). Also, major differences were found in their propensities to cause EPS. Patients treated with quetiapine were significantly less likely to experience EPS or to require anticholinergic medication in comparison to haloperidol. Patients receiving haloperidol also required higher mean doses of the anticholinergic than quetiapine, indicating a greater severity of EPS experienced by patients in the former group.

Previous reports have shown that haloperidol is associated with dose-related increases in EPS [23]. By contrast, quetiapine has been shown to have a placebo-like incidence of EPS including akathisia across its full dose range [24].

King [25] recognized EPS as a major contributor to secondary, treatment-related negative symptoms; therefore treatment with quetiapine is likely to lead to better functioning and quality of life [26]. This hypothesis was further supported by our study as more significant improvement was found in negative symptoms with quetiapine over haloperidol.

The current study’s dropout rates and average daily doses at end of trial for both haloperidol and quetiapine were inline with previous randomized clinical trials of these and other first- and second-generation antipsychotics (see [27] for a meta-analysis).

### Study limitations

The small sample size (n = 73 analyzed) and two-center study design may be regarded as limitations to our study; however, significant findings were reported only if they were consistently replicated by ANOVA analysis over group, time, and interaction.

## Conclusion

The efficacy and tolerability results of our study in hand with published studies support quetiapine for first-episode schizophrenia. Our study found quetiapine to be more efficacious and tolerable than haloperidol in this particular subset of patients for positive symptoms, negative symptoms, EPS, and fatigue; however, PANSS total scores were similar in both treatment groups. There is an inherent need for further and more long-term investigations on second-generation antipsychotics in first-episode schizophrenia.

## Abbreviations

ANOVA: Repeated measures analyses of variance; ECG: Electrocardiogram; EPS: Extra-pyramidal symptoms; DSM-IV-TR: Diagnostic and Statistical Manual of Mental Disorders, fourth edition text revision; GAF: Global Assessment of Functioning; PANSS: Positive and Negative Syndrome Scale; SAS: Simpson-Angus Scale; SD: Standard deviation.

## Competing interests

The authors declare that they have no competing interests.

## Authors’ contributions

All authors participated in the preparation of the manuscript and approved the final manuscript.

## References

[B1] Knapp M Costs of schizophrenia Br J Psychiatry 1997 171 509 518 10.1192/bjp.171.6.509 9519088

[B2] Wu EQ Birnbaum HG Shi L Ball DE Kessler RC Moulis M Aggarwal J The economic burden of schizophrenia in the United States in 2002 J Clin Psychiatry 2005 66 1122 1129 10.4088/JCP.v66n0906 16187769

[B3] Barnes TR Schizophrenia Consensus Group of British Association for Psychopharmacology Evidence-based guidelines for the pharmacological treatment of schizophrenia: recommendations from the British Association for Psychopharmacology J Psychopharmacol 2011 25 567 620 10.1177/0269881110391123 21292923

[B4] Leucht S Heres S Kissling W Davis JM Evidence-based pharmacotherapy of schizophrenia Int J Neuropsychopharmacol 2011 14 269 284 10.1017/S1461145710001380 21208500

[B5] Marder SR First and second generation antipsychotics: translating the results from pragmatic trials into clinical practice Epidemiol Psychiatr Sci 2013 22 1 2 10.1017/S2045796012000698 23388189 PMC8367325

[B6] Capehart BP Holsinger T Olanzapine on trial Am J Psychiatry 1998 155 152 author reply 153–155 9433357 10.1176/ajp.155.1.152

[B7] Mattes JA Risperidone: how good is the evidence for efficacy? Schizophr Bull 1997 23 155 161 10.1093/schbul/23.1.155 9050121

[B8] Chakos M Lieberman J Hoffman E Bradford D Sheitman B Effectiveness of second-generation antipsychotics in patients with treatment-resistant schizophrenia: a review and meta-analysis of randomized trials Am J Psychiatry 2001 158 518 526 10.1176/appi.ajp.158.4.518 11282684

[B9] Cheer SM Wagstaff AJ Quetiapine. A review of its use in the management of schizophrenia CNS Drugs 2004 18 173 199 10.2165/00023210-200418030-00004 14871161

[B10] Emsley R Oosthuizen P Evidence-based pharmacotherapy of schizophrenia Int J Neuropsychopharmacol 2004 7 219 238 10.1017/S1461145704004171 15043765

[B11] Crespo-Facorro B Perez-Iglesias R Ramirez-Bonilla M Martinez-Garcia O Llorca J Luis Vazquez-Barquero J A practical clinical trial comparing haloperidol, risperidone, and olanzapine for the acute treatment of first-episode nonaffective psychosis J Clin Psychiatry 2006 67 1511 1521 10.4088/JCP.v67n1004 17107241

[B12] Emsley RA Raniwalla J Bailey PJ Jones AM A comparison of the effects of quetiapine (‘seroquel’) and haloperidol in schizophrenic patients with a history of and a demonstrated, partial response to conventional antipsychotic treatment. PRIZE Study Group Int Clin Psychopharmacol 2000 15 121 131 10.1097/00004850-200015030-00001 10870870

[B13] American Psychiatric Association Diagnostic and Statistical Manual of Mental Disorders4th edn, text revision 2000 Washington, DC: American Psychiatric Publishing, Incorporated

[B14] Kay SR Fiszbein A Opler LA The positive and negative syndrome scale (PANSS) for schizophrenia Schizophr Bull 1987 13 261 276 10.1093/schbul/13.2.261 3616518

[B15] Piersma HL Boes JL The GAF and psychiatric outcome: a descriptive report Community Ment Health J 1997 33 35 41 10.1023/A:1022413110345 9061261

[B16] Simpson GM Angus JW A rating scale for extrapyramidal side effects Acta Psychiatr Scand Suppl 1970 212 11 19 4917967 10.1111/j.1600-0447.1970.tb02066.x

[B17] Emsley R Turner HJ Schronen J Botha K Smit R Oosthuizen PP Effects of quetiapine and haloperidol on body mass index and glycaemic control: a long-term, randomized, controlled trial Int J Neuropsychopharmacol 2005 8 175 182 10.1017/S1461145705005067 15737251

[B18] Glick ID Marder SR Long-term maintenance therapy with quetiapine versus haloperidol decanoate in patients with schizophrenia or schizoaffective disorder J Clin Psychiatry 2005 66 638 641 10.4088/JCP.v66n0515 15889952

[B19] Purdon SE Malla A Labelle A Lit W Neuropsychological change in patients with schizophrenia after treatment with quetiapine or haloperidol J Psychiatry Neurosci 2001 26 137 149 11291531 PMC1407745

[B20] Kahn RS Fleischhacker WW Boter H Davidson M Vergouwe Y Keet IP Gheorghe MD Rybakowski JK Galderisi S Libiger J Effectiveness of antipsychotic drugs in first-episode schizophrenia and schizophreniform disorder: an open randomised clinical trial Lancet 2008 371 1085 1097 10.1016/S0140-6736(08)60486-9 18374841

[B21] Abou-Setta AM Mousavi SS Spooner C Schouten JR Pasichnyk D Armijo-Olivo S Beaith A Seida JC Dursun S Newton AS Hartling L First-Generation Versus Second-Generation Antipsychotics in Adults: Comparative Effectiveness AHRQ Comparative Effectiveness Reviews 2012 Rockville (MD): Agency for Healthcare Research and Quality 23035275

[B22] Zhang JP Gallego JA Robinson DG Malhotra AK Kane JM Correll CU Efficacy and safety of individual second-generation vs. first-generation antipsychotics in first-episode psychosis: a systematic review and meta-analysis Int J Neuropsychopharmacol 2012 16 1 14 23199972 10.1017/S1461145712001277 PMC3594563

[B23] McCue RE Waheed R Urcuyo L Orendain G Joseph MD Charles R Hasan SM Comparative effectiveness of second-generation antipsychotics and haloperidol in acute schizophrenia Br J Psychiatry 2006 189 433 440 10.1192/bjp.bp.105.019307 17077434

[B24] Arvanitis LA Miller BG Multiple fixed doses of “Seroquel” (quetiapine) in patients with acute exacerbation of schizophrenia: a comparison with haloperidol and placebo. The Seroquel Trial 13 Study Group Biol Psychiatry 1997 42 233 246 10.1016/S0006-3223(97)00190-X 9270900

[B25] King DJ Drug treatment of the negative symptoms of schizophrenia Eur Neuropsychopharmacol 1998 8 33 42 10.1016/S0924-977X(97)00041-2 9452938

[B26] Riedel M Muller N Strassnig M Spellmann I Engel RR Musil R Dehning S Douhet A Schwarz MJ Moller HJ Quetiapine has equivalent efficacy and superior tolerability to risperidone in the treatment of schizophrenia with predominantly negative symptoms Eur Arch Psychiatry Clin Neurosci 2005 255 432 437 10.1007/s00406-005-0622-6 16267634

[B27] Rabinowitz J Levine SZ Barkai O Davidov O Dropout rates in randomized clinical trials of antipsychotics: a meta-analysis comparing first- and second-generation drugs and an examination of the role of trial design features Schizophr Bull 2009 35 775 788 10.1093/schbul/sbn005 18303093 PMC2696366

